# Cervical epidural abscess mimicking crowned dens syndrome

**DOI:** 10.1002/jgf2.780

**Published:** 2025-02-06

**Authors:** Nobumasa Okumura, Nana Akazawa‐Kai, Naoya Itoh

**Affiliations:** ^1^ Department of Infectious Diseases, Graduate School of Medical Sciences Nagoya City University Nagoya Aichi Japan; ^2^ Department of Infectious Diseases Nagoya City University East Medical Center Nagoya Aichi Japan

**Keywords:** epidural abscess, infective endocarditis

## Abstract

An 88‐year‐old woman was diagnosed with crowned dens syndrome. Magnetic resonance imaging showed an abscess around the odontoid process. The patient was also found to have infective endocarditis.

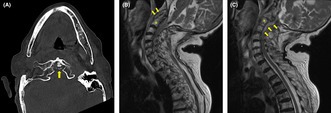

An 88‐year‐old woman with hypertension and chronic kidney disease presented with pain in her left shoulder and neck that had persisted for 2 days. She had no fever, but her pain was exacerbated by forward bending and rotation of the neck. Plain computed tomography revealed calcification of the atlantoaxial joint (Figure 1A). Therefore, she was diagnosed with crowned dens syndrome (CDS) and was prescribed acetaminophen and no antimicrobials. Five days later, she returned because of a fever and difficulty moving. She was hospitalized, and ceftriaxone was administered. Blood culture was positive for *Staphylococcus aureus* the day after admission. Cervical magnetic resonance imaging showed an abscess around the odontoid process (Figure 1B) and an epidural abscess in the spinal canal from C1 to Th2 (Figure 1C). Transthoracic echocardiography showed aortic regurgitation and vegetation adhering to the aortic valve (Figure 2). These observations led to the diagnosis of infective endocarditis.

**FIGURE 1:**
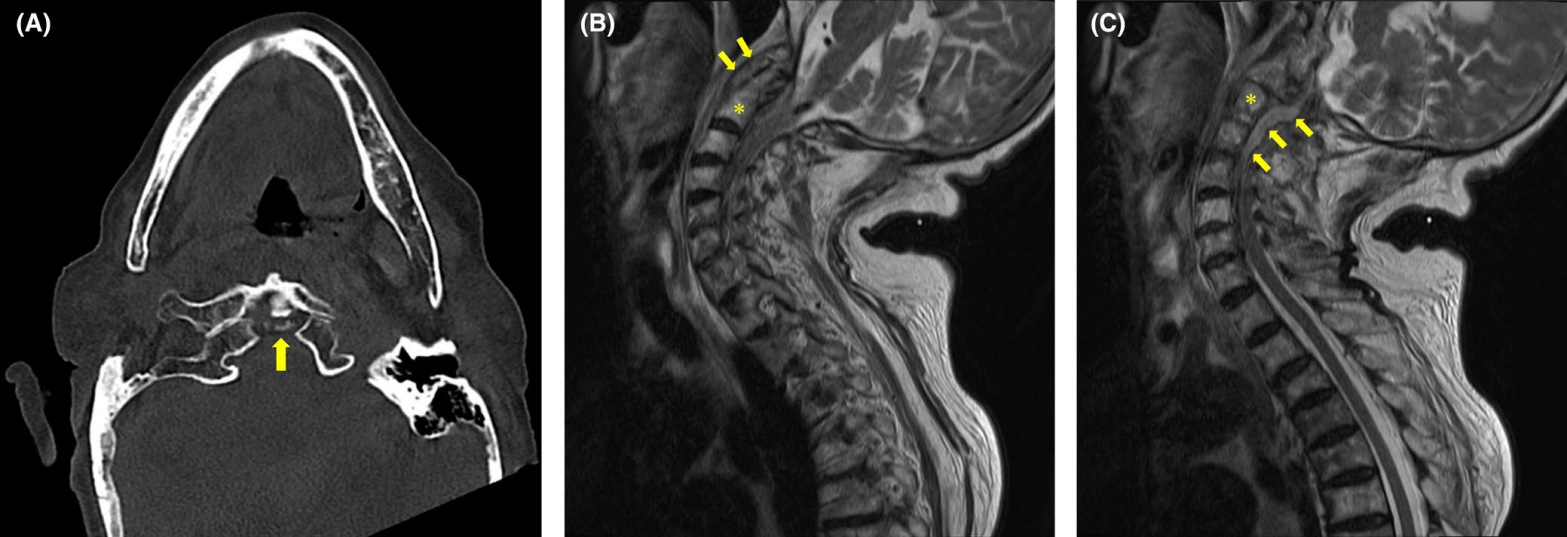
Plain computed tomography scan of the neck showing calcification of the atlantoaxial joint (arrow) (A). Cervical spine T2‐weighted magnetic resonance imaging showing an abscess around the odontoid process (B) and a spinal epidural abscess (C). The asterisk indicates the C2 vertebra.

**FIGURE 2:**
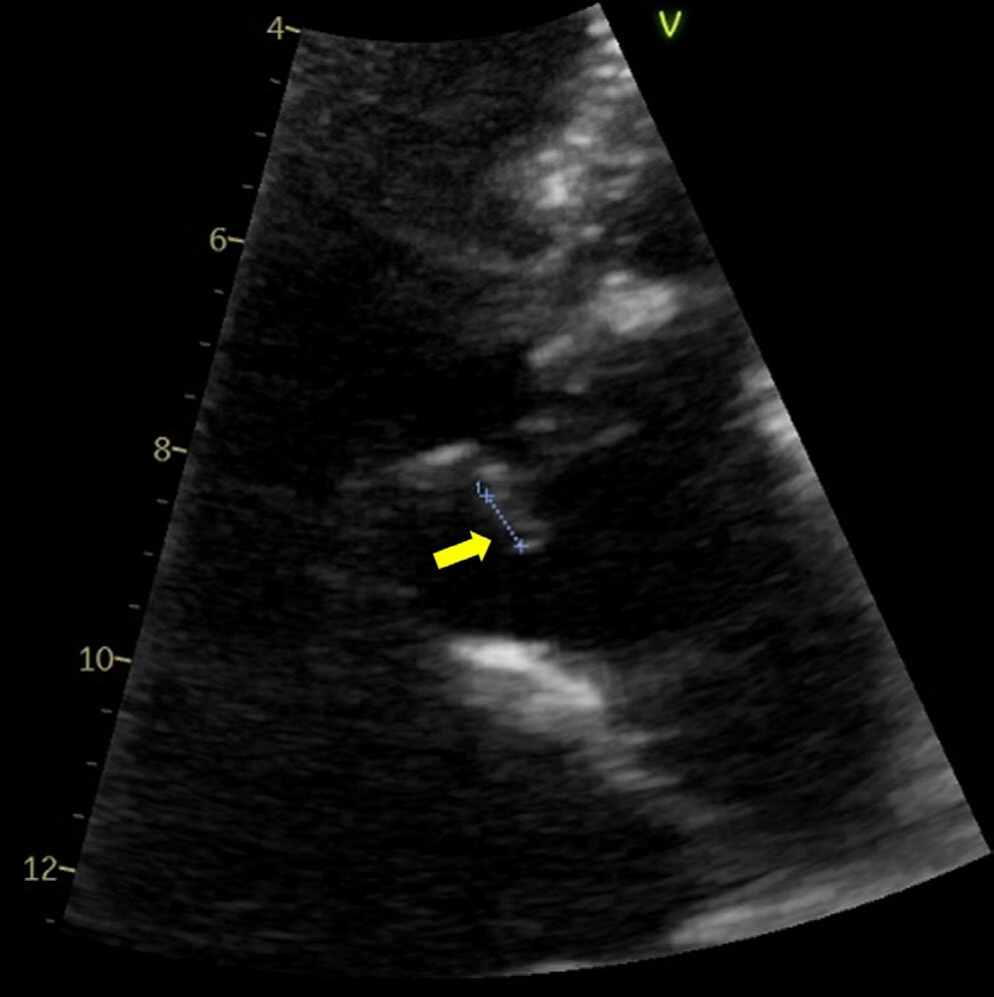
Transthoracic echocardiography showing a 5.6‐mm vegetation attached to the aortic valve (arrow).

The lumbar level is the predominant site of pyogenic vertebral osteomyelitis and epidural abscesses, whereas the cervical level is less common.^1^ Even if CDS is diagnosed based on calcification of the atlantoaxial joint, patients should be monitored closely thereafter. Therefore, clinicians should explore alternative diagnoses if the patient fails to respond to treatment for CDS. Repeat imaging studies using different modalities should also be considered because upper cervical spine lesions are difficult to differentiate from CDS,^2^ as in this case.

## AUTHOR CONTRIBUTIONS

**Nobumasa Okumura**: Conceptualization; writing—original draft preparation; writing—review and editing (lead). **Nana Akazawa‐Kai**: Writing—review and editing (supporting). **Naoya Itoh**: Funding acquisition; supervision; writing—review and editing (supporting).

## FUNDING INFORMATION

This work was supported by the Department of Clinical Infectious Diseases, Nagoya City University Graduate School of Medical Sciences, an endowment department funded by Nagoya City.

## CONFLICT OF INTEREST STATEMENT

The authors have stated explicitly that there are no conflicts of interest in connection with this article.

## ETHICS STATEMENT

Ethics approval statement: None.

Patient consent statement: Written informed consent was obtained from the patient for publication of this clinical image.

Clinical trial registration: None.
